# Supplementary material for: A unified neurocomputational bilateral model of spoken language production in healthy participants and recovery in poststroke aphasia
Source: Proc Natl Acad Sci U S A. 2020 Dec 3;117(51):32779–90. doi: 10.1073/pnas.2010193117 (PMC7768768; doi:10.1073/pnas.2010193117)
Supplement: Supplementary File [file pnas.2010193117.sapp.pdf]

Supplementary Simulations

S1. Explorations of the selection of the number of hidden units in the model

To determine the minimum number of units that were required for the model to perform the repetition task, we have developed a unilateral model with different numbers of hidden units. The selection principle followed our assumption that the key difference between the left and right pathways in the model should be quantitative, in terms of differential capacity, rather than qualitative, in terms of function. Thus we ensured that the unilateral model was capable of performing the word and nonword repetition tasks to a satisfactory level (i.e., at least 80% accuracy for both words and nonwords). The architecture of the model and the performance are illustrated in Fig. S1A.

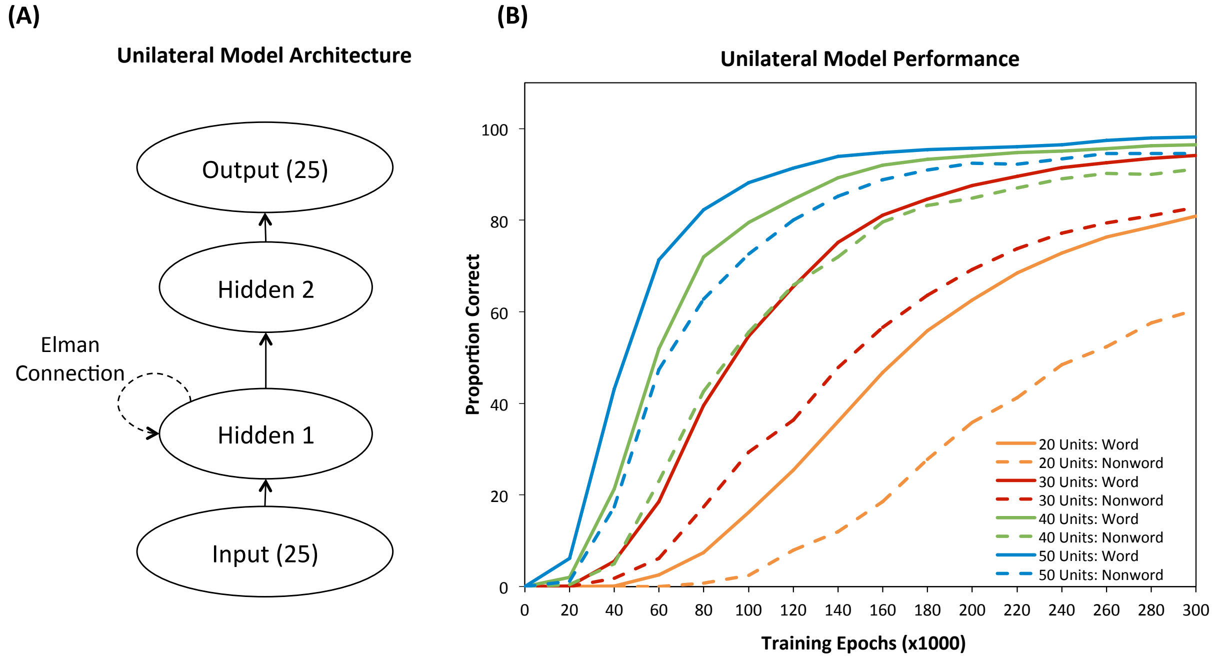

Fig. S1. (A) The architecture of the unilateral model; (B) The performance of the model with different numbers of units in hidden layers 1 and 2.

We varied the number of hidden layers 1 and 2 concurrently: 20, 30, 40, and 50. The model was trained in the same way as described in the Methods section. After 300,000 word presentations, the model was tested on both the word and nonword repetition tasks. Fig. S1B shows that the model with more hidden units can perform and generalise better. The result demonstrated that the best number of units in each hidden layer was 30 in which the model achieved about 94.1% and 82.8% accuracy on word and nonword repetition tasks respectively. This number was used for the right processing pathway in the left lateralised model reported in the main text.

S2. Explorations of the model's recovery without the implementation of inefficient learning of the surviving units after damage

To simulate behavioural patterns in post-stroke aphasia and recovery, we trained a damaged model with initial inefficient learning. This was to mimic a loss of function and activation in the damaged brain regions immediately after stroke observed in most patients (1). However, to demonstrate this implementation is not a critical determinant factor to explain different behavioural recovery patterns, we re-trained the damaged model without such an inefficiency period. It means that the surviving units in the hidden layer 1 immediately after damage can learn as efficiently as other units do in the unaffected layers. The levels of damage and the training time of recovery were the same as those described in the main text. Fig. S2 shows the recovery patterns of the damage model without initial inefficient learning in different lesion conditions.

The resulting performance and output activation patterns were broadly similar to those produced by the model with initial inefficient learning (Fig. 3). When the left lesion was mild, the activation patterns tended to return to be left lateralised during recovery. By contrast, when the left lesion was more severe the activation patterns became right lateralised, and this shift in activation led to relatively poor performance in particular for nonwords. One difference was that the transient pattern from left to right and then back to left previously observed in the left mild lesion condition

was less pronounced. However, it was clear that that activity in the right pathway rapidly increased immediately after damage with decreased activity in the left pathway, though there was no crossover. Regarding all of the other measures, the patterns were very similar to those reported in Fig. 3. These results demonstrate that the simulation without initial inefficient learning could capture the general patterns of recovery in different recovery phases. However, to better characterise the shift in activation patterns in the acute phase, the implementation of initial inefficient learning is critical in simulating a loss of function and activation in the damaged brain regions immediately after stroke observed in most patients (1).

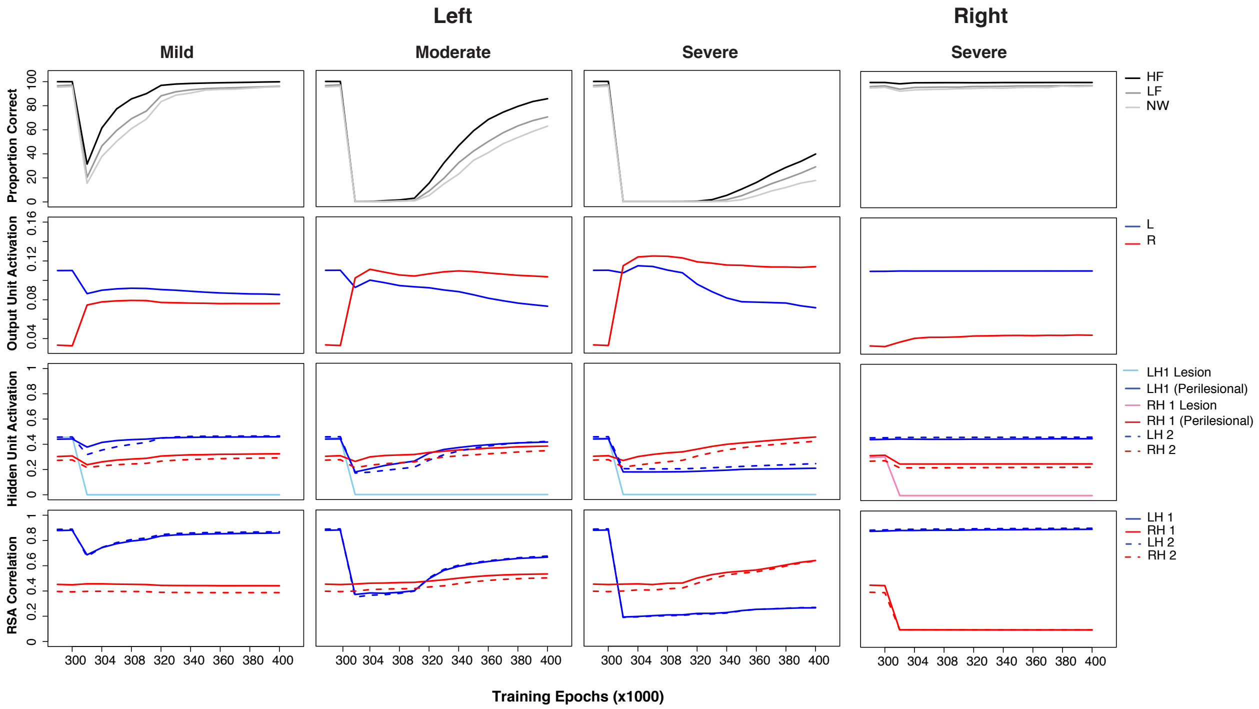

Fig. S2. Simulation patterns of post-stroke aphasia and recovery without initial inefficient training for the surviving units after damage: left mild (20%[0.2]), left moderate (50%[0.5]), left severe (80%[0.8]) and right severe (80%[0.8]) conditions. The lesion level was a combination of the proportion (%) of the units damaged and the range of noise (bracket) added to the connections to and from the hidden layer. For each lesion condition, the first panel shows model performance; the second panel shows output unit activation generated from the left and right pathway of the model separately; the third panel shows hidden unit activation for the left and right hidden layers 1 and 2.

The activations for lesioned and perilesional units are plotted separately; the last panel shows the RSA scores obtained in the left or right hidden layers 1 and 2 in the model. HF: high frequency words; LF: low frequency words; NW: nonwords; L: left; R: right; LH: left hidden layer; RH: right hidden layer.

S3. A figure for the full simulation patterns of post-stroke aphasia and recover including six measures: model performance, output unit activation, hidden unit activation, weight strength, rate of weight change, and RSA correlation

Six different measures illustrated in Figure S3 were used to reveal the underlying recovery mechanism of the damaged model. In particular, average weight strength and weight change across the hidden layers in the model were useful for understanding how the model re-learned the task during recovery and what the link was between recovery performance and re-learning processes. For instance, in the left severe lesion condition, the right output unit activation increased rather quickly after damage, and this was also reflected in an initial rise in the rate of weight change. However, performance accuracy had not started to improve at the time. When output unit activation reached a steady status, the weights continued to be updated and performance gradually improved. This may indicate two critical steps for re-learning: activation and tuning weight connections. Immediately after damage, the activation level of units in the model is generally low. Thus the first step toward re-learning is to increase the activation level and weight connections, and this is followed by re-optimising weight connections in order to re-learn the task by minimising the errors between the target and actual patterns at the output layer.

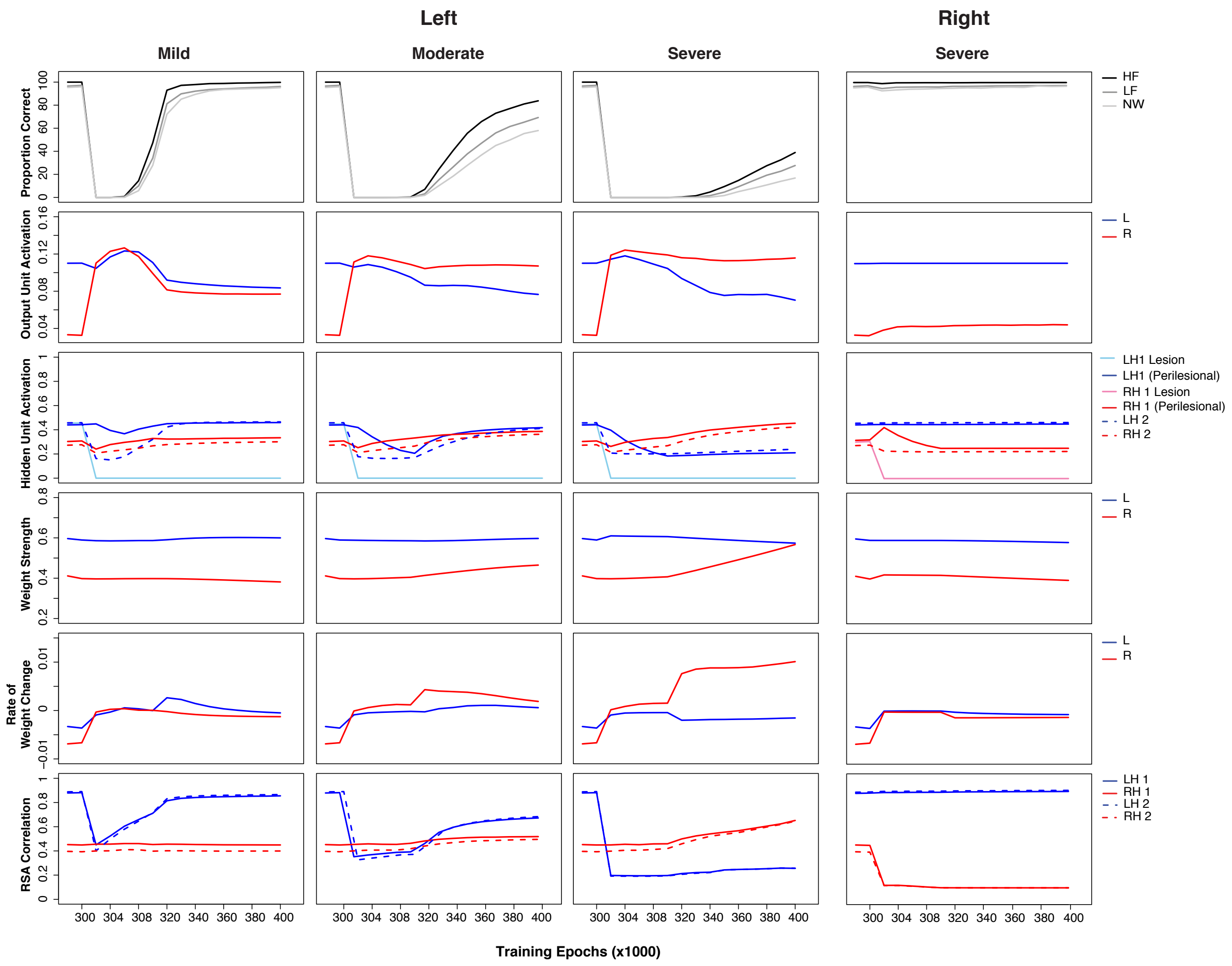

Fig. S3. The full simulation patterns of post-stroke aphasia and recovery: left mild (20%[0.2]), left moderate (50%[0.5]), left severe (80%[0.8]) and right severe (80%[0.8]) conditions. The lesion level was a combination of the proportion (%) of the units damaged and the range of noise (bracket) added to the connections to and from the hidden layer. For each lesion condition, the first panel shows model performance; the second panel shows output unit activation generated from the left and right pathway of the model separately; the third panel shows hidden unit activation for the left and right hidden layers 1 and 2. The activations for lesioned and perilesional units are plotted separately; the fourth panel shows average weight strength, averaged across all of the connections either in the left or right side of the model; the fifth panel shows the rate of weight change in strength; the last panel shows the RSA scores obtained in the left or right hidden layers 1 and 2 in the model. HF: high

frequency words; LF: low frequency words; NW: nonwords; L: left; R: right; LH: left hidden layer; RH: right hidden layer.

S4. Explorations of the inhibitory interconnectivity between the left and right hemispheres

To our knowledge, there is no direct evidence of transcallosal inhibitory connectivity outside the motor system (2-4). However, we ran an additional version of simulation with negatively constrained connections between the left and right pathways, for comparison with the model without imposed positive or negative connections (as reported in the main text; Fig. 5). The architecture of the constrained model, the training and testing procedures were identical to those reported in the main text for the unconstrained model. The only difference was that the weights of the interconnections in the constrained model were allowed to develop freely but were constrained to be negative.

Fig. S4 shows the resulting patterns produced by the constrained model with left mild, left moderate and left severe and right severe lesions, and with two different levels of interconnections (30% and 70%). The models with different levels of inhibitory interconnections produced largely similar patterns. Compared to the unconstrained model (Fig. 5), the constrained model produced more left lateralised patterns following damage and recovery, especially for the mild lesion. After a mild left lesion, the constrained model recovered to a similar accuracy level as the unconstrained model. However, in the severer lesion conditions, the constrained model produced poorer recovered performance. These results demonstrated that the constrained model was less resilient to damage. Even though the moderate and serve lesioned models produced similar activation patterns to those produced by the constrained model, the left and right pathways did not seem to work together. This observation was confirmed by the results from the right severe lesion condition, where the model did not exhibit a similar degree of impairment in the early recovery phase as that produced by the constrained model.

These results suggest that the model with imposed negative connections may have developed two independent sub-systems. If this were the case, we would anticipate small negative connections between the left and right pathways in the constrained model. Thus, we analysed the distributions of connection weights between the left and right pathways at the end of recovery. All connection weights between the left and right pathways for both hidden layers 1 and 2 were included, and they were grouped by connection direction, either from left to right or from right to left. For comparison, we also conducted the same analyses on the unconstrained model. The distributions of the interconnection weights for both the constrained and unconstrained models with two different levels of interconnections were illustrated in Fig. S5. As designed, the weights for the unconstrained model consisted of both positive and negative values, while the weights for the constrained model were all negative. More interestingly, the distribution of interconnection weights for the constrained model was much narrower than the unconstrained model. The majority of negative weights for the constrained model were very small and close to zero, and the weights decreased with lesion severity. The result indicated that as the lesion got worse, the left and right pathways worked almost independently. By contrast, the unconstrained model had larger, mixed positive and negative weights that spread around the mean (a small positive value). For the mild lesion, there were stronger weights from left to right; conversely, for the severer lesions, there were stronger weights from right to left. The result suggests that the unconstrained model was able to utilise the undamaged right pathway particularly for recovery from severer damage to the left pathway.

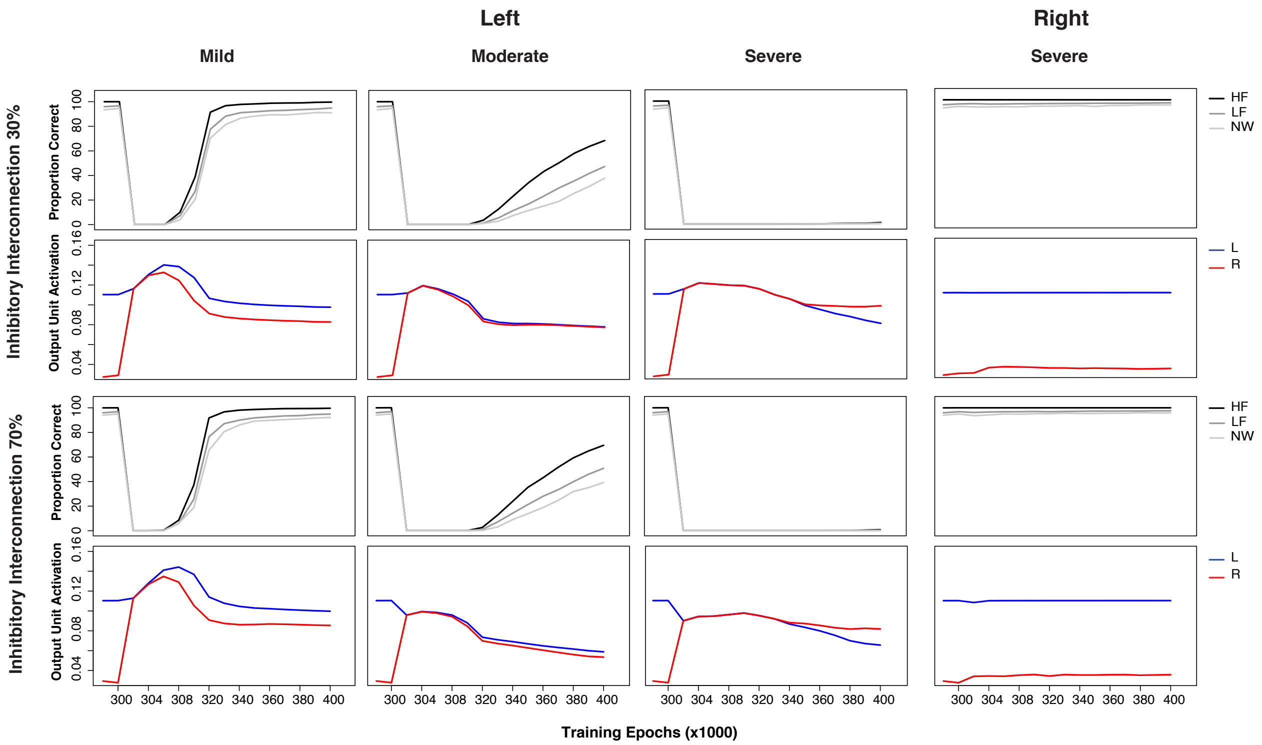

Fig. S4. Simulation patterns of post-stroke aphasia and recovery produced by the model with two levels of inhibitory interconnections (30% and 70%) between left and right sides for the left mild (20%[0.2]), left moderate (50%[0.5]), left severe (80%[0.8]) and right severe (80%[0.8]) conditions. The inhibitory connections mean that the model can develop interconnection weights freely but the values are constrained to be negative. The lesion level was a combination of the proportion (%) of the units damaged and the range of noise (bracket) added to the connections to and from the hidden layer. For each lesion and interconnection conditions, the first panel shows model performance and the second panel shows output unit activation generated from the left and right pathway of the model separately. HF: high frequency words; LF: low frequency words; NW: nonwords; L: left; R: right.

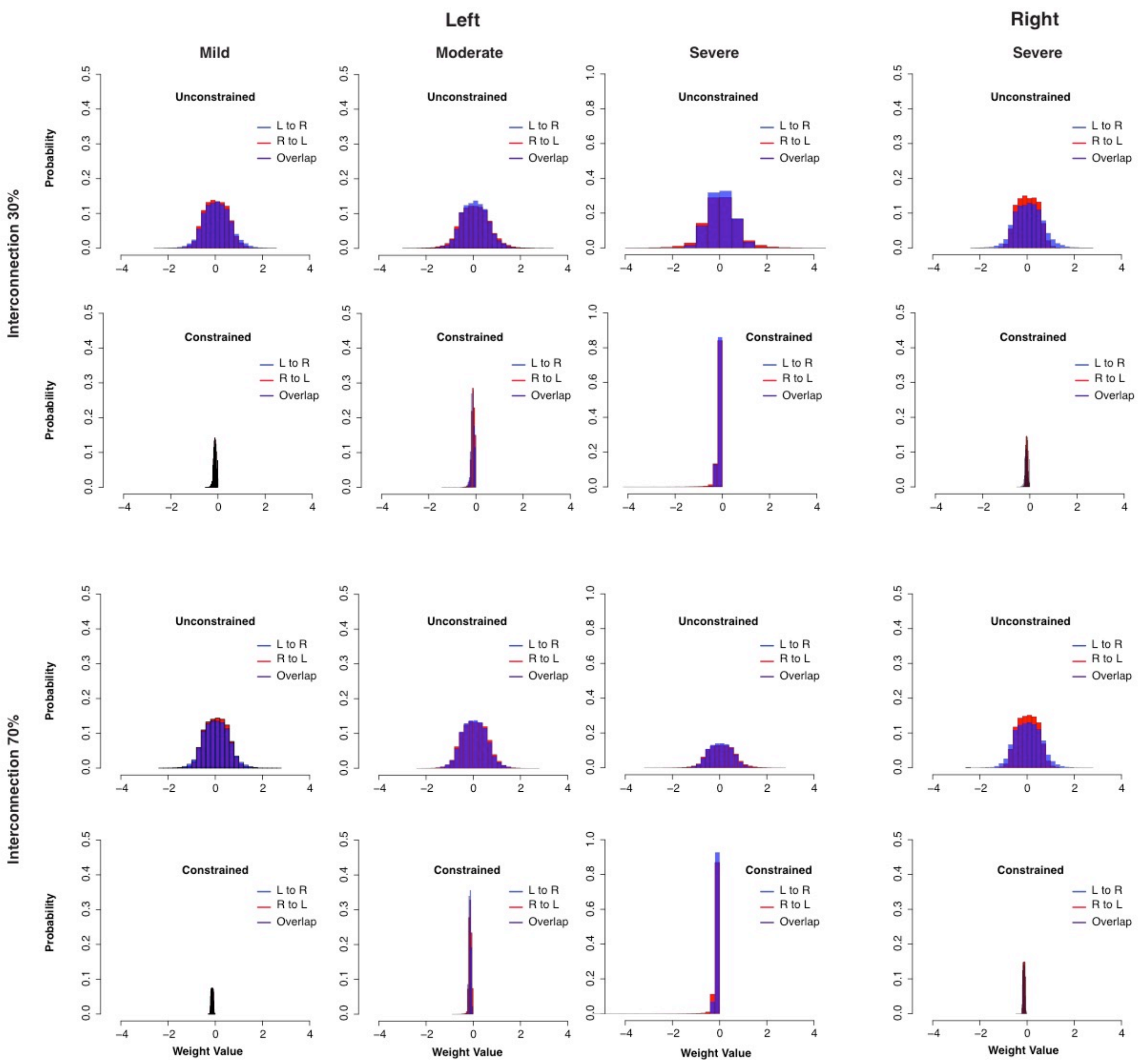

Fig. S5. The distributions of the interconnection weights for both the constrained model (interconnections constrained to be negative) and the unconstrained model (interconnections unconstrained) with two different levels of interconnections at the end of recovery for the left mild (20%[0.2]), left moderate (50%[0.5]), left severe (80%[0.8]) and right severe (80%[0.8]) conditions. The lesion level was a combination of the proportion (%) of the units damaged and the range of noise (bracket) added to the connections to and from the hidden layer. For each lesion and interconnection conditions, the first panel shows the distribution of interconnection weights for the unconstrained model and the second panel shows the distribution of interconnection weights for the

constrained model. All of the interconnection weights between the left and right pathways for both hidden layers 1 and 2 were included and they were grouped by the connection direction, either from left to right or from right to left. L to R: left to right; R to L: right to left; Overlap: overlap regions between the interconnection weights of left to right and right to left.

Supplementary Methods

M1. Phonological representations

The training set included one hundred three-phoneme high frequency and one hundred three-phoneme low frequency monosyllabic words with consonant-vowel-consonant (CVC) structures. Each word was represented in three phoneme slots, with each slot consisting of 25 phonetic features (including, voiced, nasal, labial, palatal, round, etc.). For instance, the word "*let*" its phonology was represented as l (0 0 1 0 0 0 0 0 0 0 1 0 1 0 0 0 0 0 0 0 0 0 0 0 0), ε (0 0 0 0 0 0 0 0 0 0 0 0 0 1 1 0 0 0 1 0 0 0 0 0 0 0) and t (0 0 1 0 0 0 1 0 0 0 0 0 0 0 0 0 0 0 0 0 0 0 0 0 0). The number of active phonetic features for the words in the training set ranged from 5 to 12 (M = 8.3 and SD = 1.08).

M2. Training environment

The model was trained with a learning rate of 0.01, a batch size of 1 and momentum of 0.9, using a standard back-propagation algorithm with a negative bias of -2. The sigmoid function was used as an output activation function. The weight decay was set to 0.000001. Weight connections in the model were updated after each word presentation on the basis of the cross-entropy error computed between the target and the actual activation of the output units (for details, see the following section). There was no dropout and no regularisation term. Note that a simple recurrent network generally has a sequential update procedure, which means layers in the network are updated in order. To prevent the order of update from biasing the model's reliance on one pathway, a counterbalance update sequence at the batch level was used during training.

M3. Cross-entropy error measure for back-propagation in the model

In neural network modelling, the back-propagation algorithm is often used to compute how to change connection weights in order to minimise the errors between output patterns generated by the model and their target patterns. Different error measures can be applied, that includes the summed

squared error (5) and cross-entropy error (6). The summed squared error measures the sum of squared errors across all output units:

$$E = \sum_i \left(o(i) - t(i)\right)^2$$

where *o(i)* is the output of unit *i* and *t(i)* is its target value.

Alternatively, the cross-entropy error measures Kullback-Leibler divergence (7) between the output pattern and the target pattern:

$$C = -\sum_i t(i) * log_2\big(o(i)\big) + \big(1 - t(i)\big) * log_2(1 - o(i))$$

where *o(i)* is the output of unit *i* and *t(i)* is its target value.

In our simulations, we used the cross-entropy error measure because it generates larger weight changes than the summed squared error measure. This can be particularly important when training the model with sparse representations (6), such as our phonological representations. Most of the output units were turned off by the model and it requires sufficiently large weight changes to shift its stable state for those few units that need to be on.

M4. Testing procedures

The phonological representation of each phoneme was presented sequentially for the first three time ticks. From the fourth time tick, the activation of units at the output phonological layer was recorded. If unit activation was greater than 0.5, the unit output was set to 1; otherwise, it was 0. The model's output pattern was then compared with its target representation of each phoneme from the fourth time tick to the sixth time tick sequentially. If all of the phonemes produced by the model and target phonemes were the same, then the model was judged to have spoken the word (or nonword) correctly.

Supplementary References

1. D. Saur *et al.*, Dynamics of language reorganization after stroke. *Brain* **129**, 1371-1384 (2006).
2. C. Calautti, F. Leroy, J. Y. Guincestre, J. C. Baron, Dynamics of motor network overactivation after striatocapsular stroke: a longitudinal PET study using a fixed-performance paradigm. *Stroke* **32**, 2534-2542 (2001).
3. R. S. Marshall *et al.*, Evolution of cortical activation during recovery from corticospinal tract infarction. *Stroke* **31**, 656-661 (2000).
4. N. S. Ward, M. M. Brown, A. J. Thompson, R. S. Frackowiak, Neural correlates of motor recovery after stroke: a longitudinal fMRI study. *Brain* **126**, 2476-2496 (2003).
5. M. S. Seidenberg, J. L. McClelland, A DISTRIBUTED, DEVELOPMENTAL MODEL OF WORD RECOGNITION AND NAMING. *Psychol. Rev.* **96**, 523-568 (1989).
6. D. C. Plaut, J. L. McClelland, M. S. Seidenberg, K. Patterson, Understanding normal and impaired word reading: Computational principles in quasi-regular domains. *Psychol. Rev.* **103**, 56-115 (1996).
7. S. Kullback, R. A. Leibler, On Information and Sufficiency. *Ann. Math. Statist.* **22**, 79-86 (1951).
